# Single-cell dissection of hepatocellular carcinoma immunity: from heterogeneous subtypes to precision therapeutics

**DOI:** 10.3389/fimmu.2026.1744845

**Published:** 2026-02-11

**Authors:** Yuni Liang, Hemeng Wu, Yunshan Qiu, Qiulian Mo, Peipei Chen, Mingfen Li, Hongsheng Lin

**Affiliations:** 1 Department of Clinical Laboratory, First Affiliated Hospital of Guangxi University of Chinese Medicine, Nanning, China; 2 The First Clinical Faculty of GuangXi University of Chinese Medicine, Nanning, China

**Keywords:** hepatocellular carcinoma, heterogeneity, single-cell sequencing, subsets, tumor immune microenvironment, tumor microenvironment

## Abstract

Hepatocellular carcinoma (HCC) represents one of the most prevalent malignancies worldwide and poses a critical public health challenge due to difficulties in early diagnosis, therapy resistance, and high mortality rates. The complex tumor microenvironment (TME) of HCC plays a pivotal role in tumor progression, immune evasion, metastasis, and treatment resistance. Single-cell sequencing (scRNA-seq) has emerged as a revolutionary tool for resolving the intricacies and cellular heterogeneity of the TME, with its applications in advancing therapeutic research attracting considerable attention. As the primary battleground for antitumor immune responses, the HCC tumor TME warrants comprehensive analysis of immune cell subsets at distinct developmental and functional states to elucidate the complexity of tumor immunology. This review synthesizes extensive research on TME immune cellular subpopulations, in order to summarize mainstream classifications of immune subsets at single-cell resolution and analyze their functional significance and therapeutic value through biomarker gene profiling.

## 1 Introduction

### 1.1 Epidemiology and current treatment landscape of liver cancer

Primary liver cancer represents one of the most prevalent malignant tumors worldwide and poses a significant global health challenge. Globally, it ranks as the sixth most commonly diagnosed cancer and the third leading cause of cancer-related death (1). According to 2022 global epidemiological data, approximately 865,000 new liver cancer cases and 758,000 deaths occur annually (2). As reported by China’s National Cancer Center, 368,000 new cases occurred in China in 2022, accounting for approximately 42% of the global burden and establishing the country as the most heavily impacted by liver cancer (3). A recent 2025 global projection predicts that without intervention, new annual cases will surge to 1.52 million by 2025, while deaths will rise sharply to 1.37 million (4). HCC comprising 90% of liver cancer cases, primarily arises from risk factors including hepatitis B and C infections. Notably, non-infectious etiologies such as non-alcoholic steatohepatitis linked to metabolic syndrome or diabetes are becoming increasingly prevalent causal factors in Western nations (5).Clinical management faces substantial hurdles due to limitations in early diagnosis, specifically the persistent lack of highly sensitive and specific serum biomarkers for screening (6), alongside the tendency for ultrasound and imaging modalities to miss small lesions against cirrhotic backgrounds (7). Consequently, most patients present with advanced disease at diagnosis, rendering them ineligible for curative surgery. Although tyrosine kinase inhibitors (e.g., sorafenib, lenvatinib) and immune checkpoint inhibitors(ICIs) targeting PD-1/CTLA-4 have advanced treatment paradigms, high tumor heterogeneity in HCC restricts their efficacy to subsets of patients. Frequent development of therapeutic resistance further leaves many without viable options (8, 9). The intrinsically aggressive nature of HCC which is marked by high malignancy, recurrence, and metastasis, compounds these challenges, establishing it as a major therapeutic challenge globally. Nevertheless, the rapid evolution of multi-omics technologies and intensified research into novel early diagnostic biomarkers offer promising avenues (10). Metabolites, protein signatures, genetic markers, and circulating cells represent emerging candidates to enhance early diagnostic accuracy (11, 12). Since its advent, scRNA-seq has enabled the resolution of gene expression at individual cell resolution, overcoming tissue-level heterogeneity limitations. This breakthrough allows unprecedented exploration of cellular behavior, underlying mechanisms, and tissue (13). Tumor heterogeneity is the core to HCC treatment resistance which is now being deconvoluted through scRNA-seq. By delineating the heterogeneity of the HCC tumor TME, this technology provides unprecedented insights into tumor growth, metastasis, and intercellular crosstalk mechanisms (14). Collectively, these advances offer new hope for transformative therapeutic strategies in HCC. In recent years, researchers have been attempting to summarize the roles and clinical value of various cell types in the tumor microenvironment of liver cancer (15–18). Standing on the shoulders of giants, we have synthesized a large number of review studies and experimental articles to further explore the application value of scRNA-seq in HCC. We systematically integrated the mainstream subclustering of five types of immune cells in scRNA-seq studies within the tumor microenvironment. We emphasize that the TME is not composed of static cell types, but rather a continuous spectrum of functional states that continuously shift under tumor stress, metabolic stress, and treatment exposure. Based on this foundation, we utilized a new analytical framework of marker genes - cellular developmental/functioning states - resistance mechanisms/therapeutic value translation to provide new perspectives for precise and personalized tumor interventions.

## 2 Advancements and applications of scRNA-seq technology

scRNA-seq represents a revolutionary advancement in biological research. By enabling the extraction and sequencing of RNA from individual isolated cells to obtain cell-specific expression profiles, scRNA-seq overcomes the limitations of traditional transcriptomics which only measures averaged gene expression across cell populations. This technology provides unprecedented precision in monitoring cellular activity at single-cell resolution (19). By revealing gene expression patterns in individual cells through high-throughput sequencing, scRNA-seq has been widely adopted in developmental biology, neuroscience, immunology, and oncology research (20–22).In cancer studies specifically, scRNA-seq precisely characterizes the complexity of the TME, decoding dynamic interactions among tumor cells, immune cells, and stromal cells while offering unparalleled resolution for dissecting tumor heterogeneity (23). For example, Izar et al. at Columbia University constructed multiomic single-cell atlases of untreated human melanoma brain metastases and extracranial metastases via scRNA-seq, revealing distinct TME features between these metastatic sites (24). In colorectal cancer (CRC), scRNA-seq has revealed the identification of associations between point mutations and gene expression patterns and uncovered prognostic gene biomarkers, providing critical insights into CRC’s molecular mechanisms (25, 26). A non-small cell lung cancer study leveraged large-scale single-cell profiling to uncover heterogeneity within the TME during anti-PD-1 therapy, stratifying five distinct TME subtypes with unique immune compositions and therapeutic response signatures. These findings guide clinical stratification and treatment optimization (27). Similarly, a scRNA-seq-based triple-negative breast cancer study delineated immune landscapes shaped by T-cell/B-cell crosstalk, exposing microenvironmental differences in immune cell dynamics and interactions (28).The implementation of scRNA-seq in liver cancer research is particularly extensive. Researchers recently established the largest snRNA-seq-derived hepatocyte dataset to date, capturing key cellular populations and transcriptomic profiles in HCC. This work uncovered evolutionary genomic trajectories in hepatocarcinogenesis and established a molecular classification system based on signature genes, leading to novel diagnostic frameworks and precision therapeutic strategies (29).As scRNA-seq permeates research across cancer types, it uniquely captures intercellular transcriptomic variation.scRNA-seq identifies subclonal gene expression patterns and mutational signatures, and deconstructs tissue composition via cellular proportion mapping. Combined with spatial transcriptomics, scRNA-seq-driven multiomics approaches are revealing disease progression and drug resistance mechanisms across increasingly multidimensional frameworks. These efforts concurrently identify clinically actionable biomarkers for early diagnosis and tailored therapies, establishing a new paradigm for precision medicine.

## 3 Deciphering the immune cell subsets of HCC TMEvia scRNA-seq

The immune cellular subpopulations in liver cancer comprises natural killer (NK) cells, dendritic cells (DCs), T cells, macrophages, and B cells. As a highly heterogeneous malignancy, HCC’s complex TME critically drives disease progression and therapeutic resistance by modulating tumor cell proliferation, TME promotes immune evasion and metastasis, and contributes to drug resistance mechanisms (30, 31). Throughout the oncogenic continuum, the TME fundamentally influences tumor evolution and treatment responses. Resolution of the liver cancer TME at single-cell precision allows researchers to reveal functional dynamics profiling of immune cell states, and identify key immunosuppressive cellular subsets. By analyzing functional interrogation of novel cell type-specific gene signatures, researchers reveal variations across patients and disease stages. This multifaceted approach holds far-reaching implications for overcoming current therapeutic bottlenecks and identifying novel treatment opportunities (**Supplementary Figure S1**).

### 3.1 NK cells

NK cells, as innate lymphocytes, serve as primary responders initiating immune activation. NK cells play pivotal roles in host defense and immune surveillance. These cells directly eliminate tumor cells not only through cytotoxic effector functions, but also by acting as regulatory lymphocytes, which secrete cytokines and interact with both innate and adaptive immune cells (32). Traditional classification stratifies NK cells by CD56 expression density into cytotoxicity-dominant CD56^dim^ and cytokine-secreting CD56^bright^ subpopulations. Recent studies using scRNA-seq refine this framework by delineating developmental trajectories and functional states, identifying critical subsets including cytotoxic NK cells, regulatory NK cells, and memory-like NK cells (33, 34).

Perforin-1 (PRF1), encoded by the *PRF1* gene, is a pore-forming protein stored in secretory vesicles. PRF1 acts as a key cytotoxic molecule in NK cells and plays an essential role in their tumor-killing function (35). During target cell engagement, PRF1 is released and forms pores in the target cell membrane, disrupting intracellular calcium balance. This enables Granzyme B (GZMB) and other cytotoxic agents to enter the cytoplasm, triggering an enzymatic cascade that ultimately induces tumor cell death (36). Given the critical function of cytotoxic NK cells in directly lysing cancer cells, their antitumor efficacy becomes severely limited when NK cells exhibit an exhausted phenotype. Thus, research on stimulating sustained secretion of PRF1 and GZMB by NK cells holds significant importance (37). Adoptive cell therapy using chimeric antigen receptor NK cells offers unique advantages over chimeric antigen receptor T cells therapy. It avoids triggering severe cytokine release syndrome, graft-versus-host disease, or neurotoxicity. It also permits diverse cell sourcing and enables cytotoxic enhancement through genetic engineering. Its favorable safety and efficacy profiles have been consistently validated across multiple hematologic and solid tumors (38).

Regulatory NK cells exhibit high *XCL1* gene expression, with the chemokine XCL1 being a critical component in the NK cell-mediated antitumor signaling network (39). As the primary source of XCL1, NK cells precisely recruit conventional type 1 dendritic cells (cDC1) into the TME via XCL1 secretion. This initiates a coordinated “NK-DC-T cell” immune attack axis, where cDC1 infiltration density directly dictates T-cell activation efficacy and subsequent tumor cell clearance (40). Functioning as cellular communication hubs, NK cells deficiency renders the TME an “immune-deserted island”. It permits tumor immune escape through failed immune cell activation. Notably, *XCL1* is a signature gene of immunoregulatory NK cells which is aberrantly expressed on certain tumor cells, potentially contributing to immune evasion and chemotherapy resistance (41). Given the pivotal role of the XCR1-XCL1 axis in antitumor responses, modulating NK-cDC1 crosstalk to boost cDC1 recruitment. Intratumoral XCL1 injection enhances infiltration of antigen-specific CD8^+^ T cells and NK cells, thereby suppressing tumor growth (42). XCL1 expression is significantly positively correlated with the number of tumor-infiltrating CD8^+^ T cells and PD-L1 expression, potentially inducing PD-1/PD-L1 interactions and dysfunction of CD8^+^ T cells through the XCL1-XCR1 axis, thereby predicting response to anti-PD-1/PD-L1 therapy (43). Spatial transcriptomics has confirmed the colocalization characteristics of XCL1^+^ CD8^+^ T cells with immune cells, suggesting that targeting this cell population may enhance the efficacy of immunotherapy for hepatocellular carcinoma (44).

Memory-like NK cells highly express the immunostimulatory receptor NKG2C (encoded by *KLRC2*), a type II transmembrane protein (45). NKG2C transmits activation signals upon binding HLA-E on target cells, promoting NK cell degranulation and cytokine secretion to enhance infected cell elimination. *NKG2C^+^* NK cells demonstrate potent antibody-dependent cellular cytotoxicity (ADCC) facilitating sustained clearance of infected cells. Chronic infections drive clonal expansion of *NKG2C^+^* NK cells, forming persistent “memory-like” populations with augmented effector functions (46). The competitive binding of NKG2C and the inhibitory receptor NKG2A to HLA-E dynamically regulates NK cell reactivity balance (47). In the TME, HLA-E overexpression often impairs T/NK cell effector function. To address this, novel chimeric receptors termed “NKG2A/C-swapping receptors” have been engineered. These receptors confer enhanced and specific cytotoxicity against tumors with moderate-to-high HLA-E expression while sparing normal tissues, representing a promising therapeutic strategy (48).

Current NK cell classification relies predominantly on CD56 and CD16, lacking resolution for the complexity of NK cell development and stress-responsive states. Broader tissue coverage remains limited. Integrating spatial transcriptomics can comprehensively map tissue-specific NK cell distribution and function. Future efforts should leverage epigenomic and transcriptomic datasets within spatiotemporal multi-omics approaches to dissect real-time microenvironmental regulation of NK plasticity, thereby refining subtype classification and advancing precision immunotherapies.

### 3.2 Dendritic cells

DCs represent the most potent professional antigen-presenting cells in the human immune system and serve as the exclusive activators of the naïve T cells. Functioning as critical bridges between innate and adaptive immunity, DCs play pivotal roles in initiating and sustaining T-cell antitumor responses. DCs involve in antigen presentation in tumor and lymph nodes, primary T-cell activation, and maintenance of T-cell survival and effector function within the TME. Deficiency or functional impairment of DCs severely limits antitumor immunity, thereby facilitating tumor progression (49). DCs are broadly classified into conventional DCs (cDCs, encompassing cDC1 and cDC2) and plasmacytoid DCs (pDCs).

cDCs constitute central hubs of the cancer immunity cycle. cDC1s, as master cross-presenting cells, drive CD8^+^ T cell responses against malignancies via MHC-I–restricted tumor antigen presentation,. While cDC2s predominantly prime CD4^+^ T-cell responses through MHC-II and modulate Th1/Th2/Th17/Treg immunity (50). cDC1s uniquely express *XCR1* which is the sole receptor for XCL1 enables their recruitment to inflammatory/neoplastic sites (51). Engineered T cells overexpressing XCL1 have been shown to increase DC abundance, significantly inducing antigen spreading and endogenous polyclonal T-cell responses to amplify antitumor immunity (52).

Signal regulatory protein alpha (SIRPα), a hallmark of cDC2s, interacts with CD47 to maintain immune homeostasis by restraining DC overactivation (53). *SIRPα^+^* DC2s reduce cross-presentation efficiency and impair CD8^+^ T-cell responses through CD47 signaling (54). In the TME, they mature into regulatory DCs that phagocytose apoptotic tumor cells via the AXL-SIRPα axis, upregulate PD-L1, suppress CD8^+^ T cell function, and promote Treg differentiation which critically enforcing immune tolerance. Tumor cells overexpressing CD47 evade immune clearance by engaging SIRPα on DCs to inhibit phagocytosis (55). Anti-SIRPα antibodies blocking this axis restore DC function, enhance antigen presentation and tumor phagocytosis by macrophages. Dual targeting of SIRPα and PD-L1 synergistically activates T cells, offering novel combination immunotherapy strategies (56).

pDCs, historically named for their plasma cell-like morphology, pDCs are specialized DC subsets that function as primary producers of type I interferons (IFN-I) and modulate antitumor immunity by regulating NK/T-cell activity in the TME (57). Blood dendritic cell antigen 2 (BDCA2), a C-type lectin receptor exclusively expressed on pDCs, marks this population (57). *BDCA2^+^* pDCs exhibit robust proinflammatory functions through IFN-α/TNF secretion, they can also drive immune suppression by inducing Tregs via the IDO1 pathway to dampen Th1/Th17 responses (58). Current *BDCA2* research prioritizes autoimmune diseases, focusing on IFN-I overproduction as a key pathophysiology in systemic lupus erythematosus. Therapies crosslinking BDCA2 potently inhibit IFN-I production by pDCs, presenting a promising treatment strategy for systemic lupus erythematosus (59, 60). Beyond systemic lupus erythematosus, *BDCA2* also emerges as a compelling therapeutic target for other immune disorders and hematologic malignancies.

Collectively, advances in single-cell profiling of DC subsets illuminate key mechanisms of immune regulation and therapeutic resistance in HCC.

### 3.3 T cells

T cells constitute a critical lymphocyte subset within the TME, playing a central role in antitumor immunity and serving as the foundation for most immunotherapies. CD4^+^ T cells encompass subsets including TH1, TH2, TH17, TFH, and Treg. They are all differentiated from naïve CD4^+^ T cells while each exhibiting distinct effector functions essential for shaping adaptive immune responses (61).

CC chemokine receptor 7 (CCR7), a lymph node-homing receptor expressed on dendritic cells (DCs) and T cells, guides antigen-presenting DCs and T-cell subsets to lymphoid organs via interactions with ligands CCL21/CCL19 to initiate immune responses (62). *CCR7* is highly expressed in naïve CD4^+^ T cells (63), with elevated levels observed in patients with lymph node-metastatic cancers (64). CCR7 facilitates lymphatic metastasis across malignancies as CCR7-expressing tumor cells mimic immune cell behavior by following chemokine gradients toward lymph nodes (65). Anti-CCR7 antibodies are being explored to inhibit CLL migration to lymph nodes, though clinical efficacy remains under investigation (66).

CXCR3 is a G protein-coupled receptor enriched in Th1 cells (67). CXCR3 mediates targeted migration and immune responses through ligand binding on endothelial cells, playing key roles in infections, autoimmune disorders, and tumor immunity (68). The CXCR3 axis comprises ligands CXCL9, CXCL10, and CXCL11 and shows strong cancer relevance (69). Notably, CXCR3 drives immunogenic cell death in HCC, where its expression positively correlates with T-cell infiltration. HCC patients with high CXCR3 expression display improved responses to immunotherapy (70).

CCR4, initially characterized as a TH2-selective chemokine receptor, is now recognized across multiple T-cell subsets (71). Its interaction with the atypical ligand CXCL12 drives cellular migration and proliferation in physiological and cancerous contexts (72). CXCR4 overexpression in over 20 cancer types is associated with poor prognosis and cancer stemness (73), while CCR4 antagonists suppress distant metastasis in liver cancer models (74).

The interleukin-23 receptor (*IL-23R*), primarily expressed on TH17 cells, transmits signals enhancing TH17 differentiation while promoting IL-22 secretion from CD4^+^ T cells (75, 76). IL-23R signaling induces multiple highly proinflammatory cytokines and serves as a key pathogenic driver in chronic inflammation and autoimmune diseases (77). Expanding beyond their immunological roles, TH17 cells exhibit tumor-promoting properties (78). During acute hepatic inflammation, these cells contribute to inflammatory cascades and induce HCC recurrence through dual mechanisms: facilitating cancer cell migration/invasion and enhancing tumor stemness (79).However,IL-23R involvement in this process warrants investigation.

*CXCR5* features selective expression on follicular helper T (TFH) cells. Together with its ligand CXCL13, this receptor-ligand pair governs TFH development and functional specialization (80). TFH cells are indispensable for germinal center formation and maintenance (81), thereby modulating humoral immunity. The CXCL13-CXCR5 axis contributes to tumor progression through multifaceted mechanisms involving direct tumor stimulation, immune cell recruitment, and TME modulation (82).

FOXP3 serves as the master transcriptional regulator for regulatory T cells (Tregs), orchestrating genes controlling their differentiation and immunosuppressive activity (83, 84). *FOXP3^+^* Tregs employ diverse mechanisms, including secretion of anti-inflammatory cytokines (TGF-β, IL-10, IL-35) and perforin/granzyme-mediated cytolysis (85). *FOXP3^+^* Tregs suppress antitumor immunity and facilitate tumor escape (86). Clinically, elevated *FOXP3^+^* Treg infiltration correlates with metastatic progression in HCC patients (87). Therapeutic inhibition enhances IFN-γ production in CD8^+^ T cells and restricts tumor growth (88). A recent paradigm-shifting approach demonstrates that *FOXP3^+^* -overexpressing chimeric antigen receptor T cells exhibit potent antitumor efficacy, which lack immunosuppressive functions (89).

As an immune checkpoint molecule, *CTLA-4* is expressed on activated T cells and Tregs. It both inhibits T-cell activation and delivers essential co-stimulatory signals to Tregs (90). Functionally, it suppresses immune responses through intrinsic negative regulation of effector T cells and extrinsic modulation via Treg-mediated suppression (91, 92). *CTLA-4* overexpression compromises antitumor immunity to enable immune escape (93) In HCC, *CTLA-4* blockade with neutralizing antibodies enhances tumor clearance (94), while genetic silencing augments antitumor responses (95). Therapeutically, the dual immune checkpoint regimen combining CTLA-4 inhibitor ipilimumab with PD-1 blocker *nivolumab* has achieved landmark status, which becomes China’s first approved first-line dual-immunotherapy for HCC.

CD8^+^ T cells serve as the terminal effectors of adaptive immunity, functioning as primary cytotoxic executors in antitumor immunity (96). Research focuses on their effector mechanisms, with increasing attention to CD8^+^ T cell exhaustion in tumor responses.

*LEF1* demonstrates elevated expression in naïve CD8^+^ T cells (97). Collaborating with *TCF1*, it provides sustained surveillance of CD8^+^ T cell identity and function by promoting T-lineage genes while suppressing non-T-lineage programs (98). Notably, LEF1 enhances self-renewal capacity, drug resistance, dedifferentiation, and invasiveness in HCC cells (99). For instance, it confers lenvatinib resistance by advancing Epithelial-mesenchy maltransition, migration, and invasion in HCC cells, thereby establishing *LEF1* as a novel therapeutic target to overcome acquired lenvatinib resistance (100).

Chemokine receptor *CX3CR1* express across multiple immune cell types.*CX3CR1^+^CD8^+^* T cells exhibit potent antitumor effector functions (101) and manifest exceptional cytotoxicity (102).CX3CR1 expression displays a graded transcriptional pattern reflecting CD8^+^ T cell differentiation status, with levels positively correlating with increased *GZMB*, perforin *1*, and granzyme A expression (103). While their antitumor efficacy is validated in diverse cancers, further experimental studies are required to delineate their role in HCC.

*CD69*, a C-type lectin characteristically expressed on CD8^+^ tissue-resident memory T cells, inhibits T-cell egress from tissues and participates in tissue retention alongside other lectins (104). Tissue-resident memory CD8^+^ T cells provide infection protection at barrier sites (105) and local immune defense against tumor rechallenge (106). Studies indicate that *CD69* expression modulates CD8^+^ T-cell exhaustion. Within the TME, *CD69^+^CD8^+^* T cells progressively lose effector functions, adopting an exhausted phenotype. Blocking CD69 or its signaling pathways enhances their antitumor activity (107). Notably, CD69^+^ tissue-resident memory CD8^+^ T cells expressing unique signature genes were identified in HCC patient tumors, exhibiting correlation with patient survival outcomes (108).

The granzyme K gene (*GZMK*) is enriched specifically in innate-like lymphocytes and certain CD8^+^ T-cell subsets. *GZMK^+^ T cells* typically represent central and effector memory T-cell populations (109). *GZMK^+^ T cells* display inflammatory potential, correlating positively with plasma IL-6, TNFα, and IL-8 levels (110). Recent research reveals GZMK can instigate inflammation through complement activation mediation (111). In HCC, *GZMK^+^CD8^+^ T cells* demonstrate cytotoxicity (14), and higher *GZMK* expression associates with favorable prognosis across multiple HCC cohorts (112).

Lymphocyte-activation gene 3 *(LAG3)* is expressed on CD4^+^ T cells, CD8^+^ T cells, and Tregs (113), functioning as an immune checkpoint inhibitory receptor (114). Combined LAG-3 and PD-1 signaling drives T-cell exhaustion while impeding IFN-γ-mediated autocrine antitumor immunity (115). LAG3 engagement with MHC class II and other ligands transduces T-cell inhibitory signals leading to dysfunction, which notable upregulation on exhausted T cells within the TME (116).*LAG3* correlates with poor prognosis in HCC patients when expressed at high levels (117). Whilst anti-LAG3 monotherapy yields suboptimal efficacy, combination strategies particularly with PD-1 inhibitors show superior therapeutic promise (118). Indeed, dual *LAG-3/PD-1* blockade demonstrates manageable safety and objective efficacy in advanced HCC treatment (119).

Layilin (LAYN), a transmembrane protein functioning as a C-type lectin. It participates primarily in cellular adhesion and modulates diverse including immune cell activation and T-cell subset differentiation, exerting pro-tumorigenic roles in HCC (120). *LAYN* is upregulated in tumor-infiltrating CD8^+^ T cells and Tregs within HCC and associates with their suppressive functions (97). *LAYN*-overexpressing CD8^+^ T cells display hallmark exhaustion features and diminished antitumor capacity. Patients with high *LAYN* levels exhibit poorer overall survival (121).

T-cell subset profiling remains a sustained research focus. Despite considerable efforts on CD8^+^ T cells due to their prominence in tumor immunity, this field nears investigational saturation with diminishing novel discoveries. Emerging paradigms now emphasize exploring untapped CD4^+^ T cell subsets. Notably, Tregs—whose discoverers received the *2025 Nobel Prize in Physiology or Medicine*,show exceptional promise as a prime candidate for next-generation investigations. Their discovery underscores the transformative potential of regulatory T cells in cancer immunology.

### 3.4 Macrophages

Landmark advances in macrophage functional studies originated from the 1990s-established M1/M2 polarization model. In this model, IFN-γ/LPS-induced classically activated M1 macrophages exhibit pro-inflammatory/antitumor functions, while IL-4/IL-13-induced alternatively activated M2 macrophages display anti-inflammatory/pro-repair activities. This paradigm, inspired by Th1/Th2 differentiation concepts, significantly advanced our understanding of macrophage roles in tumorigenesis and infections (122), revealing polarization’s centrality in immune regulation. However, scRNA-seq and spatial transcriptomics have exposed limitations in this binary model. It oversimplifies *in vivo* macrophage heterogeneity, depicts context-dependent polarization states, and fails to incorporate developmental origin diversity. The M1/M2 polarization framework has essential differences in conceptual assumptions and research methods compared to single-cell scRNA-seq. The M1/M2 polarization model originates from *in vitro* stimulation experiments. It assumes that macrophages differentiate into two relatively stable, opposing functional states under specific stimuli (e.g., IFN-γ/LPS or IL-4/IL-13) to explain immune regulation during inflammation or repair processes (123). However, this framework implies the premises of discrete states and single dominant signals, making it difficult to reflect the true cellular landscape shaped by multiple overlapping signals, metabolic reprogramming, and developmental origin differences in complex *in vivo* microenvironments (124). In contrast, scRNA-seq is a data-driven, hypothesis-free single-cell analysis method. By simultaneously measuring the expression levels of thousands of genes at single-cell resolution, it directly reveals the continuous spectrum of macrophage functional states, dynamic transition trajectories, and spatial correlations within tissues (125, 126). At the methodological level, M1/M2 relies on a limited number of markers and artificially defined polarization conditions, while scRNA-seq characterizes a multidimensional, highly heterogeneous network of transcriptional states through clustering analysis, pseudotime inference, and cell communication modeling. Contemporary research integrates multi-dimensional approaches including single-cell subpopulation definition, spatial functional mapping, and pseudotime trajectory inference (127, 128), providing insights beyond static classifications. Current representative categories include inflammatory macrophages, angiogenic macrophages, interferon macrophages, regulatory macrophages, lipid-associated macrophages, and tissue-resident macrophages (124).

*CCL3* (macrophage inflammatory protein-1α, *MIP-1α*), a CC-chemokine engaging CCR1/CCR5/CCR9 receptors, is highly expressed by macrophages upon inflammatory stimulation (129). This pro-inflammatory chemokine actively recruits monocytes/macrophages to inflammatory sites (130). In human HCC, CCL3 orchestrates antitumor immunity, including enhancing phagocytic activity, upregulating MHC molecules for antigen presentation, recruiting/activating T cells, and reprogramming the TME to restore adaptive immunity (131). Therapeutic CCL3 administration enhances TME immunogenicity. Mice with CCL3-enriched tumors showed delayed growth post anti-PD-1 monoclonal antibody treatment, suggesting combinational αPD-1/CCL3 strategies may augment clinical efficacy (132).

Secreted phosphoprotein 1 (SPP1), a pleiotropic phosphoglycoprotein, regulates immune responses while promoting tumor proliferation, invasion, and therapy resistance (133). Although produced in multiple organs, SPP1 expression is restricted to osteoblasts, fibroblasts, macrophages, dendritic cells, lymphocytes, and monocytes. Cancer cells also express *SPP1*, and elevated circulating SPP1 or tumor SPP1 correlates with poor prognosis across cancers (134). *SPP1^+^* macrophages promote tumor angiogenesis and hypoxic microenvironments by secreting extracellular matrix proteins (e.g., CD44, MMPs) and activating HIF-1 signaling (122). In CRC, *SPP1^+^* macrophage/FAP^+^ fibroblast interactions through TGF-β and IL-17 signaling exacerbate pro-angiogenic and immunosuppressive milieus. *SPP1^+^* TAMs across tumor types overexpress angiogenesis-related genes (e.g., VEGF pathway members) and correlate with adverse outcomes (135). Compared with normal tissues, tumors exhibit significantly enriched *SPP1^+^* macrophage infiltration. High *SPP1* expression induces resistance to anti-PD-L1 immunotherapy (122) and potentiates liver cancer stemness, growth, migration, and chemoresistance (136). Mechanistically, SPP1^-^CD44 interactions activate multiple exhaustion-associated pathways in CD8^+^ T cells, with SPP1 transcriptionally regulating T cell exhaustion (137), thereby facilitating tumor immune evasion. High expression of SPP1 is significantly associated with poor prognosis in various cancers, including colorectal cancer and liver cancer. Anti-SPP1 combined with PD-1 inhibitors (e.g., NCT05230901) can reverse the immunosuppressive functions of TAMs (138). SPP1^+^ TAMs secrete MMP9 and collagen, promoting tumor invasion and metastasis, and the expression level of SPP1 can predict resistance to PD-1 therapy in esophageal squamous cell carcinoma (139). Despite the enormous potential of SPP1 as a therapeutic target in cancer, fibrosis, and other diseases, and with several drug discovery projects underway, there are currently no approved SPP1-targeted drugs globally.

ISG15 (Interferon-Stimulated Gene 15) encodes the first identified ubiquitin-like protein modifier, functioning both as a free intracellular/extracellular molecule and as a post-translational modifier during ISGylation (140). During viral infections, IFN-I induction is a key component of innate immunity which triggers *ISG15* expression, which participates in cancer cell apoptosis via IFN production (141). While *ISG15* upregulation enhances IFN-mediated macrophage phagocytosis and antiviral activity (142), tumor-secreted *ISG15* acts as a TME factor that induces M2-like polarization, promotes tumor progression, and suppresses cytotoxic T-lymphocyte responses (143). Consequently, ISG15 exhibits context-dependent roles in cancer. It functions as either a tumor suppressor or oncogene by altering distinct biological pathways across cancer types. Elevated *ISG15* expression in HCC cell lines and clinical specimens correlates with cell cycle progression, cancer cell proliferation/migration, and poor 5-year survival (144).

Arginase 1 (Arg1), encoded by the *ARG1* gene, hydrolyzes arginine as a rate-limiting enzyme of the urea cycle. Produced by tumor-infiltrating myeloid cells including macrophages, granulocytes, DCs, immature progenitors. High Arg1 activity depletes L-arginine in TME, suppressing T-cell proliferation and reducing effector T-cell populations (145). Macrophage polarization toward M2 phenotypes involves metabolic reprogramming in which *Arg1* plays key roles (146). *Arg1*-expressing macrophages inhibit CD4^+^ T-cell proliferation/cytokine production (147), thereby supporting tumor growth. Notably, tumor cells supply arginine to macrophages to enhance polyamine biosynthesis, driving immunosuppressive polarization and CD8^+^ T-cell dysfunction (148). Targeting Arg1 effectively reprograms macrophages; Arg1-derived peptide vaccines activate *Arg1*^+^ CD4^+^ T cells, promoting inflammatory macrophage phenotypes and generating antitumor immune responses to inhibit growth (149).

Apolipoprotein E (APOE), a plasma cholesterol-transport protein and Alzheimer’s disease (AD) risk factor, has been predominantly studied in AD pathology (150). Highly expressed in macrophages, macrophage-derived APOE exerts immunomodulatory functions beyond lipid transport (151). Macrophage *APOE* critically regulates extracellular vesicle production, influencing T-lymphocyte proliferation, activation, and IFN-γ secretion (152). Enriched in M2 macrophages, exosomal APOE mediates macrophage-gastric cancer crosstalk to promote metastasis (153). *APOE^+^* macrophages interact with exhausted CD8^+^ T cells, attenuating immune checkpoint inhibitor efficacy, whereas *APOE* blockade enhances immunotherapy responses (154). Pan-cancer analyses reveal differential *APOE* expression between tumors and normal tissues, correlating with clinical phenotypes and serving as a prognostic biomarker across cancers (155). APOE is a strong genetic risk factor associated with Alzheimer’s disease, and APOE genotyping can be used for risk stratification and early diagnosis (156). APOE may serve as a biomarker for HER2-negative breast cancer, aiding in predicting responses to immunotherapy. APOE^+^ TAMs promote the expression of genes related to lipid metabolism and are associated with an immunosuppressive tumor microenvironment and combination immunotherapy may enhance anti-tumor immune responses (157). While research on APOE has primarily focused on Alzheimer’s disease, its role in tumor biology remains highly controversial and faces multiple methodological challenges.

LYVE-1 (Lymphatic Vessel Endothelial Hyaluronan Receptor-1), a marker of lymphangiogenesis, is expressed on lymphatic endothelial cells and macrophages (158). Associated with tumor lymphangiogenesis and TME modulation (159), macrophage-expressed LYVE-1 mediates leukocyte docking/transmigration (160) and cancer cell-endothelial adhesion. In melanoma metastasis studies, *LYVE-1* blockade enhanced pro-inflammatory states in pre-metastatic livers, altering TME to reduce hepatic metastasis (161). *LYVE-1^+^* macrophages represent a protumorigenic, anti-inflammatory subset promoting hyaluronan remodeling in TME, and their depletion delayed mammary tumor growth (162). Anti-LYVE-1 monoclonal antibodies inhibit lymphangiogenesis, suppressing both primary tumor formation and metastasis, highlighting *LYVE-1* as a promising therapeutic target (163).

Although the M1/M2 paradigm remains influential, growing evidence urges its abandonment in single-cell contexts (164, 165). This binary model is established from *in vitro* polarization—oversimplifies tissue macrophage states, ignoring their continuous, dynamic, and multidimensional *in vivo* landscapes. Persisting with rigid classifications obscures critical biological insights and impedes precision therapeutics. As scRNA-seq becomes increasingly accessible, multidimensional interrogation of functional subsets will provide comprehensive blueprints for tumor immunotherapy.

### 3.5 B cells

B cells, long recognized as core components of the adaptive immune system, play pivotal roles within the TME. Beyond antibody secretion, antigen presentation, and cytokine production, they modulate immune responses to influence tumor progression (166). Based on developmental trajectories and functional states, major B-cell subsets include naïve B cells, memory B cells, germinal center (GC) B cells, plasma cells, and regulatory B cells (Bregs) (167, 168).

*TCL1A*, a marker of naïve B cells, regulates signaling during early B-cell development; its downregulation signifies B-cell maturation (169). TCL1A promotes proliferation by activating stem cell expansion pathways (170), enhancing *KI67* expression and modulating the cell cycle (reducing G1 phase, increasing S/G2 phases). It also upregulates *CR2* expression on B cells (154), enabling efficient antigen presentation (155). Notably, TCL1A promotes tertiary lymphoid structure formation (171), which correlates with positive immunotherapy responses and survival outcomes (172–174), underscoring its value as a therapeutic target.

*CD27*, a member of the TNF receptor superfamily and key memory B-cell marker, interacts with its ligand CD70 to regulate humoral immunity and tolerance (175). CD27 sustains memory B-cell survival by activating NF-κB signaling to inhibit apoptosis and guiding migration to secondary lymphoid organs for survival factors. Upon antigen re-exposure, CD27-CD70 engagement activates NF-κB and MAPK/ERK pathways, driving rapid memory B-cell activation and proliferation. Subsequently, CD27 signaling induces plasma cell differentiation via downstream transcriptional activators, facilitating high-affinity antibody secretion during secondary immune responses (176). The CD27-CD70 axis represents a promising target across hematologic and solid tumors: the agonistic anti-CD27 antibody varlilumab shows clinical efficacy (177), soluble CD27 predicts anti-PD-1 monotherapy response (178), and serves as an HCC risk biomarker (179).

The transcriptional repressor *BCL6*, predominantly expressed in GC B cells, is essential for GC formation and B-cell differentiation (180). By repressing differentiation-related genes, it maintains GC B cell proliferation/survival and governs TFH-dependent somatic hypermutation/class-switch recombination, ultimately generating high-affinity antibody-secreting plasma cells and memory B cells (181). BCL6 also acts as an HBV promoter suppressor in hepatocytes, enhancing chemokine production and immune cell liver infiltration (182). However, *BCL6* is oncogenic: it epigenetically represses pro-apoptotic genes via promoter binding to promote cancer cell survival (183), making its targeted degradation therapeutically valuable (184).

*MZB1* (Marginal Zone B and B1 cell-specific protein 1), upregulated during plasma cell differentiation (185), encodes an ER-resident co-chaperone critical for antibody secretion capacity (186). *MZB1* deficiency impairs plasmablast migration (via β1-integrin dysregulation) and terminal plasma cell differentiation (187). Overexpressed in hematologic malignancies, *MZB1* sustains malignant B-cell protein folding and chemoresistance in multiple myeloma (188, 189). As a key regulator of ER homeostasis and antibody production, *MZB1* targeting may modulate plasma cell function to mitigate inflammation (190).

Regulatory B cells (*Bregs*) suppress immunity primarily through IL-10 production (191). *CD38*high Bregs secrete IL-10/TGF-β to convert naïve TH cells into FoxP3^+^ Tregs (192), while suppressing antigen-specific T-cell effector functions and Th17 differentiation (193),collectively facilitating tumor immune evasion. CD38 governs multiple inflammatory processes (migration, adhesion, phagocytosis, antigen presentation/release) and is a therapeutic target in hematologic malignancies (194). Anti-CD38 antibodies (e.g., isatuximab, daratumumab) induce antibody-dependent cytotoxicity, with clinical efficacy validated in multiple myeloma, NK-cell lymphoma, and CD19^-^ B-cell malignancies (195, 196).

While B-cell biology in hematologic tumors is well-characterized, their roles in solid tumors (breast, lung, colorectal cancers) remain limited. B-cell subsets exhibit context-dependent plasticity across tumor types, disease stages, and treatments. Current insights represent only partial functional delineations; deeper investigations into TME-specific mechanisms and subset-targeted immunomodulation are warranted.

### 3.6 Perspectives on scRNA-seq-driven cell subtype dissection

In the cascade of antitumor immune responses, which spanning antigen presentation, phagocytosis, cytotoxicity, and antibody secretion. NK cells, DCs, T cells, macrophages, and B cells each execute distinct roles in tumor cell elimination. However, this process is frequently hijacked by tumors to facilitate their growth and immune evasion. Beyond these, neutrophils, endothelial cells, and fibroblasts also exert critical functions within the TME. Critically, these cell types are not independent entities; they engage in intense intercellular crosstalk through diverse signaling networks. Thus, elucidating cell-cell communication is paramount for decoding TME complexity. Research on cell interactions supported by scRNA-seq is no longer limited to average signals from specific cell types (197); it can precisely identify individual cells’ gene expression, protein secretion, and signaling characteristics, thereby highlighting cellular heterogeneity (198). The integration of spatial transcriptomics further reveals the spatiotemporal dynamics and functional diversity of these interactions. Researchers have used scRNA-seq and spatial transcriptomics to discover that MRC1^+^ tumor-associated TAMs interact with cancer-associated fibroblasts through WNT5A and HGF signaling to promote the metastasis of hepatocellular carcinoma (124). Microfluidic chip analyses combined with scRNA-seq secretion profiling showed that only a subset of CD8^+^ T cells secretes IFN-γ after contacting tumor cells, while most cells enter an exhausted state due to PD-1/PD-L1 signaling (199).Additionally, researchers found through ligand-receptor analysis that NK cells recruit cDC1 to the tumor core by secreting XCL1 and CCL5, forming a positive feedback loop that enhances anti-tumor immunity (200). In summary, single-cell technologies have transformed the study of cell interactions from a blurry average relationship to a finely regulated network with spatial, temporal, and state resolution. While not all discussed cell subtypes have been extensively studied in HCC, mechanisms observed in other cancers may exhibit comparable or opposing effects in HCC which finds awaiting further experimental validation. Meanwhile, we have listed the subgroups and marker genes discussed in detail in **Table 1** and some potential subgroup marker genes that are not mentioned in **Table 2**. scRNA-seq enables precise characterization of transcriptional profiles that define cellular states. Yet “signature gene” expression is inherently multifaceted: genes may be expressed across multiple cell types or exhibit functional reversals during different developmental stages of the same lineage. Under pathological stresses, which including tumor hypoxia, inflammatory stimulation, metabolic stress, or therapeutic exposure—virtually any functional gene may be induced, silenced, or reprogrammed, thereby sculpting novel cellular states with unique phenotypic and biological significance. The exploration of functional subtypes seems to hold near-limitless potential (**Supplementary Figure S2**).

**Table 1:** Immune cell subtype marker genes and clinical applications.

Marker Gene	Clinical Applications	References
NK Cells		
XCL1	Intratumoral XCL1 injection recruits cDC1 cells, boosting antigen-specific CD8^+^ T cell infiltration	(42)
NKG2C	NKG2A/C switch receptor engineered to target HLA-E-overexpressing tumors	(48)
DCs		
XCR1	Engineered XCL1-overexpressing T cells enhance DC-mediated antitumor immunity	(52)
SIRPα	Anti-SIRPα antibody blocks CD47-SIRPα axis to improve DC antigen presentation efficiency	(56)
BDCA2	Litifilimab targets BDCA2 to inhibit IFN-α production by pDCs (treating systemic lupus erythematosus)	(59)
T cells		
CXCR3	CXCR3-high expression positively correlates with T cell infiltration and predicts immunotherapy response	(70)
CCR4	CCR4 antagonists suppress HCC distant metastasis	(74)
CTLA-4	Ipilimumab plus nivolumab becomes China's first dual-checkpoint inhibitor regimen for first-line HCC treatment	(94)
LAG3	LAG3-PD-1 bispecific antibody (relatlimab) demonstrates objective response in advanced HCC	(119)
FOXP3	FOXP3-overexpressing CAR-T cells enhance antitumor efficacy (preclinical)	(89)
Macrophages		
CCL3	CCL3 synergizes with anti-PD-1 to enhance antitumor efficacy (preclinically validated)	(132)
SPP1	SPP1^+^ TAMs cause resistance to PD-L1 blockade,serve as detrimental prognostic biomarkers	(122, 137)
APOE	APOE inhibitors potentiate checkpoint inhibitor efficacy	(154)
B cells		
BCL6	Targeted degradation of BCL6 for B-cell lymphoma therapy	(184)
CD38	Daratumumab/isatuximab treat multiple myeloma via ADCC mechanisms	(192, 196)
CD27	Soluble CD27 (sCD27) as biomarker for PD-1 therapy response and HCC risk prediction in HCV-sustained virological response patients	(179)

**Table 2:** Supplementary marker genes for immune cell subtypes unlisted in main text.

Marker Gene	Biological Function	References
NK Cells		
GZMB	GZMB serves as a core cytotoxic effector molecule in NK cells, inducing target cell apoptosis through caspase pathway activation upon release from cytoplasmic granules.	(209)
TIGIT	TIGIT overexpression significantly impairs IFN-γ and TNF production, diminishing NK cell cytotoxic function against tumor targets. It induces NK cell dysfunction and reduces immune response capacity.	(210)
CD160	CD160 engages both classical and non-classical MHC class I molecules, thereby triggering NK cell cytotoxicity and cytokine production to promote NK cell activation and degranulation; conversely, CD160 is essential for efficient IFN-γ production in activated NK cells	(211)
DCc		
BATF3	Collaborates with IRF8 to support cDC1 precursor development and maturation. Drives effector gene expression programs enabling cDC1-specific molecules required for cross-presentation and T cell co-stimulation.	(212, 213)
IRF8	Orchestrates cDC1 development and function, thereby enhancing tumor antigen cross-presentation competence and potentiating antitumor immunity.	(214)
T Cells		
GNLY	Synergizes with perforin and granzymes to directly eliminate pathogens/tumor cells upon release by T/NK cells, while augmenting inflammatory responses.	(215)
CD6	Costimulatory receptor on T cells that binds ALCAM on APCs and epithelia, delivering activation signals to T lymphocytes.	(216, 217)
CXCR6	The CXCR6+CD8+ T cell subset exhibits enhanced activation and cytotoxicity; as a principal responder population mediating immune checkpoint therapeutics, intratumoral CXCR6+CD8+ T cells are indispensable for driving cancer regression.	(218)
CXCL13	The CXCL13:CXCR5 axis orchestrates intercellular interactions to modulate lymphocyte infiltration in the tumor microenvironment, thereby dictating tumor responsiveness to cytotoxic and immune-targeted therapeutics; concurrently, CXCL13 drives recruitment of CXCR5+CD8+ T cells to the liver—a subset critically enabling enhanced viral control in chronic hepatitis B infection.	(219, 220)
Macrophages		
VEGFA	Promotes angiogenesis and tissue repair; functionally linked to tumor progression. Function description pending validation. (Note: Source paper describes FAP^+^-SPP1^+^ interactions)	(122)
FOLR2	Marks protumoral M2-like macrophages that drive immunosuppression via DC recruitment and CD4^+^FOXP3^+^ Treg differentiation.	(221)
B Cells		
MKI67	Essential for B cell proliferation/differentiation. Ki-67 overexpression facilitates rapid cell cycle entry post-antigen stimulation, generating plasma/memory B cells for humoral immunity.	(222)

## 4 Discussion

HCC remains a global health challenge due to high incidence, mortality, and treatment resistance. Despite diagnostic advances, the complex TME severely limits therapeutic efficacy. scRNA-seq has revolutionized our understanding of HCC TME heterogeneity, mapping immune cell dynamics and cellular interactions. These insights reveal spatiotemporal complexity and communication networks, guiding precision treatment strategies and target discovery. This review synthesizes scRNA-seq advances in profiling HCC TME immune architecture, including T cells, B cells, macrophages, NK cells, and dendritic cells. The technology surpasses bulk RNA limitations to map antitumor (e.g., *GZMK^+^* T, *CCL3*^+^) and protumor subsets (Tregs, Bregs, suppressive). Key regulatory mechanisms identified involve chemokine receptors (CXCR3), checkpoints (LAG-3), functional modulators (FOXP3/SPP1), and transcription factors (BCL6) that govern immune function, treatment resistance, and metastasis. These findings reveal high-value therapeutic targets beyond diagnostic utility. Although scRNA-seq has significantly improved our ability to analyze the heterogeneity of the HCC TME, there are still clear limitations in its studies. Differences in tissue dissociation and transcript capture efficiency may lead to the underestimation of certain immune subsets and the omission of key signaling genes (201). Additionally, conventional scRNA-seq only provides static transcriptional snapshots, making it difficult to accurately reflect the spatiotemporal dynamics of immune cells during tumor progression and treatment responses (202). The results of single-cell clustering are highly sensitive to algorithms and thresholds, leading to a lack of standardized definitions for subsets across different studies, and transcript levels do not always equate to functional states (203). Therefore, single-cell discoveries still require integration with spatial genomics, multi-omics approaches, and functional and clinical validations to achieve reliable mechanistic explanations and clinical translation (201, 204).The development of spatially resolved transcriptomics (SRT) significantly complements the loss of spatial information in scRNA-seq. The combined application of scRNA-seq and SRT technologies provides unprecedented solutions for studying the functional genomics of individual cells and their spatial environments within tissues (205). High-resolution techniques, such as Stereo-seq and 10× Visium HD, enhance spatial resolution to subcellular levels through *in situ* capture technology, allowing for the direct localization of gene expression at tumor boundaries and within heterogeneous regions (203). SRT enables near-complete transcriptome analysis (>20,000 genes) on formalin-fixed paraffin-embedded samples, identifying the spatial exclusion of PD-L1^+^ TAMs and CD8^+^ T cells, thereby revealing potential mechanisms of resistance to immunotherapy (206). Researchers have constructed tumor cell lineage states, clonal structures, and spatial maps of the tumor microenvironment by analyzing primary and metastatic tumor samples from patients, discovering significant transcriptional differences in tumors between primary sites and various metastatic sites (207). In these studies, SRT technology not only retains single-cell precision but also reconstructs the spatial context of tissues, making it an indispensable complementary tool in cancer exploration. Clinical translation studies targeting modulation of the TME remain at a nascent stage. From a clinical perspective, while scRNA-seq has revealed the high heterogeneity of the HCC immune microenvironment, it still faces practical challenges in explaining mechanisms of immune therapy resistance and guiding patient stratification. In patients resistant to immune ICIs, immunosuppressive myeloid cell subsets, functionally exhausted T cell states, and abnormal cellular communication networks are often enriched. However, these characteristics exhibit significant individual variance among different patients, making it difficult to translate them into unified, actionable resistance biomarkers. Additionally, the cell subsets and gene features defined by scRNA-seq are primarily retrospective associations and lack simplified indicators that are strongly correlated with clinical outcomes and detectable in routine pathology or liquid biopsies, thereby limiting their practical application in patient stratification and treatment decisions. Therefore, there is an urgent need for future efforts to combine single-cell analyses with clinical. Critically, research disproportionately prioritizes T cells and macrophages, overlooking pivotal contributions from B, NK, and dendritic cells. Moving forward, research should prioritize multi-omics integration using spatial transcriptomics coupled with scRNA-seq to construct “spatial-cell type-functional state” atlases of HCC TME. Longitudinal tracking via TCR/BCR sequencing and epigenetics will elucidate temporal evolution of immune subsets during tumor progression/therapy. Developing rational combination therapies, such as immune ICIs with Treg/TAM/Breg-targeting agents, CAR-T/NK against impaired molecules (BCL6, SPP1), or cell therapies combined with cytokines (XCL1) remains essential. AI-driven analytics will enhance scRNA-seq data processing, phenotype identification, and biological network interpretation, enabling predictive treatment-response models for true personalized therapy. While T cell/macrophage research nears saturation, focused efforts on understudied B/NK/DC subsets hold untapped potential. By establishing a single-cell-level prognostic model to predict targeted therapy responses based on cancer patients’ cellular subtypes, we will significantly improve the precision of personalized treatment (208).Through sustained innovation and cross-disciplinary collaboration, the profound biological understanding derived from single-cell technologies will ultimately herald a new era of precision immunotherapy in HCC—delivering transformative survival benefits for patients worldwide.

## Supplementary material

Supplementary Figure S1 Architecture of the TME.

Supplementary Figure S2 Antitumor immune response orchestrated by immune cells. Legend: ①NK cells serve as the "primary initiators" of antitumor immunity. The loss or downregulation of MHC class I molecules on tumor cells fails to deliver inhibitory signals to NK cells. Instead, NKG2D activation receptors recognize and bind stress-induced ligands (e.g., MICA/B) on malignant cells, triggering cytotoxic responses. Activated NK cells release perforin-1 and granzyme B to directly lyse tumor cells. Additionally, they secrete CCL5 and XCL1, guiding cDC1 recruitment for optimal tumor antigen uptake and cross-presentation. A subset of NK cells differentiates into memory-like NK cells, exhibiting enhanced antibody-dependent cellular cytotoxicity (ADCC) for long-term tumor surveillance②Tumor cells express mutated neoantigens or tumor-associated antigens (TAAs). cDC1 captures tumor antigens via pattern-recognition receptors (PRRs, e.g., TLRs), internalizes them, and processes peptides onto MHC class I for CD8+ T cell priming. Meanwhile, cDC2 presents antigens via MHC class II to activate CD4+ T cells, orchestrating broader immune coordination.③Macrophages engulf tumor cells via PRR recognition, followed by antigen processing and MHC class I/II presentation to CD8+ and CD4+ T cells, respectively. This bridges innate and adaptive immunity.④Upon recognizing MHC I-presented tumor antigens, CD8+ T cells differentiate into cytotoxic T lymphocytes (CTLs). CTLs eliminate tumors via perforin-1/granzyme B-induced apoptosis and generate long-lived memory CD8+ T cells for rapid recall responses.Activated by MHC II-antigen complexes, CD4+ T cells differentiate into Th1 subsets, secreting IFN-γ to enhance CTL function, or Th2 cells, which support B cell responses.⑤BCR engagement triggers tumor antigen internalization, processing, and MHC II presentation to CD4+ T cells. T-B cell interaction via CD40-CD40L and Th2-derived cytokines (e.g., IL-4) drive affinity-matured antibody production by plasma cells. Concurrently, memory B cells are generated for secondary responses. Antibodies tag tumor cells for complement-mediated lysis and NK/macrophage-mediated ADCC, amplifying tumor clearance.
