# Correction: Single-cell dissection of hepatocellular carcinoma immunity: from heterogeneous subtypes to precision therapeutics

**DOI:** 10.3389/fimmu.2026.1817873

**Published:** 2026-03-06

**Authors:** Yuni Liang, Hemeng Wu, Yunshan Qiu, Qiulian Mo, Peipei Chen, Mingfen Li, Hongsheng Lin

**Affiliations:** 1 Department of Clinical Laboratory, First Affiliated Hospital of Guangxi University of Chinese Medicine, Nanning, China; 2 The First Clinical Faculty of GuangXi University of Chinese Medicine, Nanning, China

**Keywords:** hepatocellular carcinoma, heterogeneity, single-cell sequencing, subsets, tumor immune microenvironment, tumor microenvironment

“Supplementary Figure 1 Architecture of the TME” and “Supplementary Figure 2 Antitumor Immune Response Orchestrated by Immune Cells” were omitted. The file(s) have now been published.

The original version of this article has been updated.

