# Supplementary material for: Single-cell dissection of hepatocellular carcinoma immunity: from heterogeneous subtypes to precision therapeutics
Source: Front Immunol. 2026 Feb 11;17:1744845. doi: 10.3389/fimmu.2026.1744845 (PMC12932472; doi:10.3389/fimmu.2026.1744845)
Supplement: Supplementary file 3 [file DataSheet1.pdf]

①NK cells serve as the "primary initiators" of antitumor immunity. The loss or downregulation of MHC class I molecules on tumor cells fails to deliver inhibitory signals to NK cells. Instead, NKG2D activation receptors recognize and bind stress-induced ligands (e.g., MICA/B) on malignant cells, triggering cytotoxic responses. Activated NK cells release perforin-1 and granzyme B to directly lyse tumor cells. Additionally, they secrete CCL5 and XCL1, guiding cDC1 recruitment for optimal tumor antigen uptake and cross-presentation. A subset of NK cells differentiates into memory-like NK cells, exhibiting enhanced antibody-dependent cellular cytotoxicity (ADCC) for long-term tumor surveillance②Tumor cells express mutated neoantigens or tumor-associated antigens (TAAs). cDC1 captures tumor antigens via pattern-recognition receptors (PRRs, e.g., TLRs), internalizes them, and processes peptides onto MHC class I for CD8+ T cell priming. Meanwhile, cDC2 presents antigens via MHC class II to activate CD4+ T cells, orchestrating broader immune coordination.③Macrophages engulf tumor cells via PRR recognition, followed by antigen processing and MHC class I/II presentation to CD8+ and CD4+ T cells, respectively. This bridges innate and adaptive immunity.④Upon recognizing MHC I-presented tumor antigens, CD8+ T cells differentiate into cytotoxic T lymphocytes (CTLs). CTLs eliminate tumors via perforin-1/granzyme B-induced apoptosis and generate long-lived memory CD8+ T cells for rapid recall responses.Activated by MHC II-antigen complexes, CD4+ T cells differentiate into Th1 subsets, secreting IFN-γ to enhance CTL function, or Th2 cells, which support B cell responses.⑤BCR engagement triggers tumor antigen internalization, processing, and MHC II presentation to CD4+ T cells. T-B cell interaction via CD40-CD40L and Th2-derived cytokines (e.g., IL-4) drive affinity-matured antibody production by plasma cells. Concurrently, memory B cells are generated for secondary responses. Antibodies tag tumor cells for complement-mediated lysis and NK/macrophage-mediated ADCC, amplifying tumor clearance.
